# Clinical effect of T-SPOT.*TB* test for the diagnosis of tuberculosis

**DOI:** 10.1186/s12879-019-4597-8

**Published:** 2019-11-21

**Authors:** Yanfen Ma, Ruicheng Li, Jinghui Shen, Longmei He, Ying Li, Ning Zhang, Qian Wu, Jinling Zhang, Jie Zheng, Xiaoqin Wang

**Affiliations:** 1 Department of Clinical Laboratory, The First Affiliated Hospital of Xi’an Jiaotong University, Xi’an, 710061 Shaanxi Province China; 2 Department of Clinical Laboratory, The Second Affiliated Hospital of Air Force Medical University, Xi’an, 710038 Shaanxi Province China; 3 Department of Clinical Laboratory, Xi’an Central Hospital, Xi’an, 710003 Shaanxi Province China; 4 Department of Clinical Laboratory, Shaanxi Provincial Hospital of Traditional Chinese Medicine, Xi’an, 710061 Shaanxi Province China; 5 Department of Clinical Laboratory, Shaanxi KangFu Hospital, Xi’an, 710061 Shaanxi Province China; 6 Department of Clinical Laboratory, Xi’an Encephalopathy Hospital, Xi’an, 710061 Shaanxi Province China; 7 Clinical Research Center, The The First Affiliated Hospital of Xi’an Jiaotong University, Xi’an, 710061 Shaanxi Province China

**Keywords:** T cell spot test of tuberculosis infection, *Mycobacterium tuberculosis*, Active tuberculosis, Clinical diagnosis

## Abstract

**Background:**

The goal of this study was to further investigate the clinical effectiveness of the T-SPOT.*TB* test in diagnosing tuberculosis (TB), including the effects of T-SPOT.TB test on evaluating diverse TB types and locations.

**Methods:**

We collected 20,332 specimens from patients suspected to have TB. Afterwards, we performed an integrative analysis of T-SPOT.*TB* results and clinical diagnoses, and evaluated the composition ratio and positive detection rate of the T-SPOT.*TB* test in various age groups, sample types, and hospital departments. In addition, we compared the spot number and composition rate between latent TB infection (LTBI), active TB infection, and old TB infection groups. The active TB group was then further divided into pulmonary TB (PTB), pulmonary and extrapulmonary TB (PETB), and extrapulmonary TB (EPTB) subgroups, and we evaluated whether there were statistical differences in spot number and composition rate between subgroups.

**Results:**

Positive results from the T-SPOT.*TB* test were found across different age groups, specimen types, and hospital departments. Elderly patient groups, pleural effusion samples, and thoracic surgery departments showed the highest rates of positivity. There were no statistically significant differences in spot number of CFP-10 and ESAT-6 wells between disease groups or active TB subgroups. The composition rate, however, was significantly different when ESAT-6 and CFP-10 wells were double-positive. The spot number and composition rate were statistically different between the three disease groups, but showed no significant differences between the three subgroups of active TB.

**Conclusions:**

The results of T-SPOT. *TB* test showed differences in LTBI, active TB and old TB. Additionally, a higher spot number level was observed in the active TB group.

## Background

Tuberculosis (TB) is a common chronic disease caused by infection with the facultative intracellular pathogenic bacteria *Mycobacterium tuberculosis*, and is a serious danger to public health [1]. It has been reported that *Mycobacterium tuberculosis* (*M.TB*) can spread hematogenously to various tissues and organs including the lung apices, lymph nodes, spleen, and liver, which has been considered to be one crucial infectious source in TB occurrence [2]. Remarkably, there are approximately 10.4 million new cases of TB and 1.8 million deaths every year around the world [3] and nearly one-third of the population in the world is regarded to be latently infected, according to the World Health Organization’s investigation and assessment [4]. Recently, China has been reported to be 1 of 30 high-burden TB countries, due to high TB morbidity [5]. In addition, TB characteristics tend to be concealed by complications and other symptoms because of the growing numbers of elderly patients, increasing patients with drug resistance and extensive immune impairment, causing the slow progression of TB, atypical symptoms and misdiagnosis or missed-diagnosis in clinical examinations [6]. Therefore, it is essential to control TB progression, and undertake early prevention, diagnosis, and treatment.

Acid-fast staining, TB antibody examination and real-time quantitative PCR have been the predominant methods for *M. tuberculosis* detection in clinical analyses [7]. Unfortunately, these approaches have numerous drawbacks, such as unsatisfactory sensitivity and specificity, high specimen requirements, and susceptibility to immune state [8]. Encouragingly, the T-SPOT.*TB* assay (an interferon (IFN)-γ release assay) is based on detecting secreted IFN-γ in *M. tuberculosis-*specific T-cells stimulated by *Mycobacterium*-specific antigens. These antigens include early secreted antigenic target 6 (ESAT-6) and culture filtrate protein 10 (CFP-10), which have been successfully utilized in T-cell effect tests to determine whether *M. tuberculosis* infection exists [9, 10]. Moreover, accumulating evidence has suggested that this powerful approach can effectively diagnose *M. tuberculosis* infection for patients with or without TB symptoms, and has relatively high sensitivity and specificity [11]. However, a T-SPOT.*TB* analysis based on a larger sample size has rarely been performed in TB diagnoses, and an evaluation of the T-SPOT.*TB* test’s effects on diverse TB types and locations has not been undertaken.

Here, 20,332 subjects suspected of having TB were enrolled at the First Affiliated Hospital of Xi’an Jiaotong University, and subjected to T-SPOT.*TB* assay. Subsequently, corresponding comparison analyses including the composition ratio and positive rate in various age groups, sample types, and hospital departments were performed. Moreover, the spot number and composition rate in different disease types (latent TB, active TB, and old TB), and three active TB subgroups (PTB, PETB and EPTB subgroups) were evaluated. This work will contribute to carrying out efficient TB clinical diagnosis and prevention based on T-SPOT.*TB* test.

## Methods

### Subjects

In total, 20,332 samples were collected from patients suspected of having TB. The participants’ average age was 53.15 ± 18.16 years, and they were admitted to the First Affiliated Hospital of Xi’an Jiaotong University from July 2013 to May 2017. Specifically, there were 11,453 males between 6 months and 96 years of age (Mean age: 54.70 ± 18.45), and 8879 females between 3 months and 96 years of age (Mean age: 52.53 ± 17.71 years). All patients were enrolled according to the following criteria: 1) the subjects were without heart, liver, or kidney diseases, and did not have an HIV infection; 2) patients were suspected to have TB; 3) Patients were not taking any therapies that involved immunosuppression or enhancer medication. The exclusion criteria included: 1) cases lost to follow-up; 2) cases lost to death; 3) cases that were not diagnosed as the TB at end points.

Informed consent was obtained from all participants (informed consent of patients under 16 years of age was obtained from their guardians), and this study was approved by the ethics committee of the First Affiliated Hospital of Xi’an Jiaotong University (NO. XJTU1AF2018LSK-161).

### Diagnostic criteria for different groups

In light of the diagnostic criteria for pulmonary TB outlined by the Ministry of Health of the People’s Republic of China, the included patients were grouped as follows: 1) LTBI: tuberculin test was positive and patients who had no history of the Bacillus Calmette – Guerin (BCG) vaccination; or the test result of T-SPOT.TB was positive and there was no clinical manifestation of TB and corresponding evidence from etiology, pathology and imageology. 2) active TB: this required that tubercle bacilli were detected from bacterial cultures or sputum smear, that caseate or Langhans’ giant cells were observed by pathological examination, and that antituberculous therapy was effective with relevant imaging support. Moreover, active TB was further divided into three subgroups based on pathological site: pulmonary TB (PTB), pulmonary and extrapulmonary TB (PETB), and extrapulmonary TB (EPTB). 3) old TB: this required that subjects with a history of TB were healed, but pathological changes were found according to the imageological diagnosis, that symptoms of TB poisoning were not observed and tests from etiology and pathology were negative, and that the patients with these characteristics were diagnosed with “other diseases”.

### Sample collection and handling

Peripheral venous blood samples of 5 ml were obtained from patients suspected of having TB using heparin lithium-anticoagulant tubes. Mononuclear cells were then isolated to prepare a cell suspension. Finally, the T-SPOT.*TB* assay (Oxford Immunotec, Ltd., Abingdon, UK) was performed as follows: briefly, the cell suspension was seeded onto T-SPOT.*TB* plates and incubated with ESAT-6 (specific antigen), CFP-10 (specific antigen), positive control, or negative control, respectively. A 100 μl cell suspension was then added into corresponding microwells, and these were cultured in an incubator with 5% CO_2_ at 37 °C. Microwells were then washed four times with phosphate buffer solution (PBS), before 50 μl of secondary antibody solution was added into each well, and the assay was incubated for 1 h at 2–8 °C. Subsequently, 50 μl of chromogenic agent was added, and the plate was processed under light avoidance for 7 to 12 min before termination with distilled water. The number of spots were measured, where one spot represented one T cell which could secrete specific cytokines [12]. The final results interpretation was in accordance with following criteria: 1) Results were considered positive in two scenarios: first, if the number of spots in the negative control group was less than 6, and the spot number in CFP-10 or ESAT-6 wells was 6 spots greater than that of negative control. Second, if the spot count in the negative control group was 6 to 10, and the CFP-10 or ESAT-6 spot number was more than two times that of the negative control. 2) Results were considered negative if the spot number did not meet the above criteria, and the positive control performed normally. Finally, we performed statistical analyses of spot number and, the spots accounted for the proportion of total spots (composition rate) in different disease types (LTBI, active TB and the old TB) and different active TB subgroups (PTB, PETB and EPTB).

### Statistical analysis

All data analyses were carried out using SPSS software (version 18.0, IBM). A χ^2^ test was applied to assess comparison analyses. And “inspection rate” (means the percentage of certain samples among all tested samples) was calculated. T-SPOT.*TB* results showed a skewed distribution and were expressed as the median and interquartile range. Nonparametric tests were used for the comparative analyses of different groups, and *p* < 0.01 was considered statistically significant.

## Results

### Distribution of T-SPOT.TB results in different ages

A total of 20,332 patients were distributed into various age groups, of which the childhood group had the fewest individuals (0–6 years old; 68 cases), while the overwhelming majority of patients belonged to the middle-aged group (41–65 years old; 9735 cases). Interestingly, the positive rate of tests was highest in the elderly group (41.91%, Table 1). In addition, positive rate of whole blood was 36.60% (Table 1).

**Table 1:** The results of T-SPOT.*TB* in 20,332 patients from different age groups and specimen types

Items	Cases	Proportion (%)	Positive cases	Proportion (%)	Positive proportion (%)	Positive rate (%)
Age groups						
0–6 years old	68	0.33	3	0.01	0.04	4.41
7–17 years old	410	2.02	65	0.32	0.87	15.85
18–40 years old	4247	20.89	1441	7.09	19.31	33.93
41–65 years old	9735	47.88	3494	17.18	46.81	35.89
≥ 66 years old	5872	28.88	2461	12.10	32.97	41.91
Total	20,332	100.00	7464	36.71	100.00	36.71
Specimen types						
Whole blood	20,107	98.89	7361	36.20	98.61	36.60
Total	20,332	100.00	7464	36.71	100.00	36.71

### Distribution of T-SPOT.TB results in different hospital departments

The samples selected in this work covered 29 hospital departments, such as respiration, rheumatology, or urology departments (Table 2). We noted that the highest inspection rate was in the respiration department (8261 cases; 40.63%), followed by the rheumatology department (2924 cases; 14.38%). The pain department exhibited the lowest test rate (10 cases; 0.05%). Additionally, in terms of positive rates of tests, the thoracic surgery department was in the lead (53.20%), followed by the general surgical department (50.00%). The lowest positive rate of tests was 5.26% for the pediatric department.

**Table 2:** The results of T-SPOT.TB in 20,332 patients from different clinical departments

Departments	Cases	Proportion (%)	Positive cases	Propotion (%)	Positive proportion (%)	Positive rates (%)
Pain	10	0.05	4	0.02	0.05	40.00
Cardiovascular surgery	25	0.12	9	0.04	0.12	36.00
Ear-nose-throat	39	0.19	19	0.09	0.25	48.72
Emergency	47	0.23	13	0.06	0.17	27.66
General surgery	50	0.25	25	0.12	0.34	50.00
Gynaecology and obstetrics	51	0.25	24	0.12	0.32	47.06
Breast Surgery	53	0.26	19	0.09	0.25	35.85
Traditional Chinese medicine	75	0.37	33	0.16	0.44	44.00
Abdominal tumor surgery	79	0.39	32	0.16	0.43	40.51
Peripheral vascular	79	0.39	37	0.18	0.50	46.84
Radiotherapy	83	0.41	38	0.19	0.51	45.78
Dermatology	84	0.41	26	0.13	0.35	30.95
Hepatobiliary surgery	99	0.49	39	0.19	0.52	39.39
Pediatrics	152	0.75	8	0.04	0.11	5.26
Urinary surgery	157	0.77	77	0.38	1.03	49.04
Central ICU	180	0.89	46	0.23	0.62	25.56
Medical oncology	208	1.02	69	0.34	0.93	33.17
Endocrinology	358	1.76	154	0.76	2.07	43.02
Orthopedics	365	1.80	172	0.85	2.31	47.12
Cadre’s ward	381	1.87	167	0.82	2.24	43.83
Cardiology	478	2.35	214	1.05	2.87	44.77
Neurology	518	2.55	187	0.92	2.51	36.10
Thoracic surgery	594	2.92	316	1.55	4.24	53.20
Hematology	734	3.61	170	0.84	2.28	23.16
Digestive System	778	3.83	304	1.50	4.08	39.07
Infectious	1203	5.92	377	1.85	5.06	31.34
Nephrology	2267	11.15	853	4.20	11.44	37.63
Rheumatology	2924	14.38	705	3.47	9.46	24.11
Respiration	8261	40.63	3327	16.36	44.57	40.27
Total	20,332	100.00	7464	36.71	100.00	36.71

### Comparisons of spots numbers and composition rate in three groups

The findings of T-SPOT.*TB* tests suggested that there were 7464 positive cases, out of a total of 20,332 cases. After removing undiagnosed (691 cases) and non-whole blood specimens (103 cases), the remaining 6760 cases were divided into three groups, including LTBI (4612 cases), active TB (1814 cases), and old TB groups (334 cases). The spot number types of T-SPOT.*TB* tests were as follows: ESAT-6 unique positive, CFP-10 unique positive; ESAT-6 and CFP-10 double positive; total spot number of ESAT-6 and CFP-10. A comparative analysis implied that the spot number of ESAT-6 and CFP-10 wells were not significantly different between each disease group (*p* > 0.05), but the composition rate in each group was dramatically different when ESAT-6 and CFP-10 microwells were double positive (*p* < 0.01) (Table 3). For intergroup comparative analyses, we found that the spot number and composition rate were statistically different among three groups (*p* < 0.01). CFP-10 and ESAT-6 double positive rates in active TB (79.16%) were higher than LTBI (56.15%) and old TB (66.77%) (Table 3).

**Table 3:** Comparative results of the numbers of spots and composition in three groups

Spot types	LTBI	Active TB infection	Old TB infection	χ^2^/*P*	Z/*P*
ESAT-6 unique positive					
Cases	1281	270	69		
Composition rate	27.78	14.88	20.66	120.844/< 0.001	
Median spot number	12.00	20.00	16.00		57.080/< 0.001
Spot number interquartile range	8.00,23.00	12.00,36.25	8.50,26.50		
CFP-10 unique positive					
Cases	741	108	42		
Composition rate	16.07	5.96	12.57	116.473/< 0.001	
Median spot number	11.00	17.00	19.50		19.355/< 0.001
Spot number interquartile range	7.00,20.00	9.00,31.75	7.00,32.25		
ESAT-6 and CFP-10 double positive					
Cases	2590	1436	223		
Composition rate	56.15	79.16	66.77	297.397/< 0.001	
ESAT-6/median spot number	24.00	44.00	29.00		320.640/< 0.001
ESAT-6/ Spot number interquartile range	12.00,46.00	24.00,80.00	14.00,60.00		
CFP-10/ median spot number	24.00	46.00	26.00		320.728/< 0.001
CFP-10 (Spot number interquartile range)	12.00,47.00	23.00,89.00	15.00,54.00		
Total spot number					
Cases	4612	1814	334		
Composition rate	100.00	100.00	100.00		
ESAT-6/ median spot number	15.00	36.00	20.00		635.730/< 0.001
ESAT-6/(Spot number Interquartile range)	7.00,33.00	17.00,70.00	9.00,43.00		
CFP-10/median spot number	12.00	35.00	19.00		535.277/< 0.001
CFP-10 (Spot number interquartile range)	4.00,30.00	11.00,75.00	6.00,40.25		

TB: tuberculosis; LTBI: latent TB infection

### Comparisons of spot numbers and composition rate in three subtypes of active TB

The active TB group contained three subgroups (PTB: 547 cases; PETB: 199 cases; EPTB: 1068 cases) as mentioned above. The results revealed that the spot number for CFP-10 and ESAT-6 microwells in each subgroup were not significantly different (*p* > 0.05), but the composition rate in each subtype was significantly different when CFP-10 and ESAT-6 microwells were double positive (*p* < 0.01). Moreover, there were no significant differences in terms of the numbers of spots, and the composition rate among three subgroups (*p* > 0.05) (Table 4).

**Table 4:** Comparative results of the numbers of spots and composition in three subgroups

Spot types	PTB	PETB	EPTB	χ^2^/*P*	Z/*P*
ESAT-6 unique positive					
Cases	89	27	154		
Composition rate	16.27	13.57	14.42	1.284/0.526	
Median spot number	22.00	21.00	17.00		5.655/0.059
Spot number interquartile range	14.00,42.00	12.00,39.00	10.00,31.50		
CFP-10 unique positive					
Cases	27	5	76		
Composition rate	4.94	2.51	7.12	7.798/0.020	
Median spot number	18.00	9.00	16.50		0.014/0.993
Spot number interquartile range	9.00,30.00	8.50,125.00	9.25,33.75		
ESAT-6 and CFP-10 double positive					
Cases	431	167	838		
Composition rate	78.79	83.92	78.46	3.091/0.213	
ESAT-6/ Median spot number	44.00	42.00	44.00		1.516/0.469
ESAT-6/(Spot number interquartile range)	25.00,83.00	24.00,87.00	23.00,78.00		
CFP-10/ Median spot number	42.00	53.00	47.00		3.337/0.189
CFP-10 (Spot number interquartile range)	22.00,86.00	29.00,94.00	23.00,89.00		
Total spot number					
Cases	547	199	1068		
Composition rate	100.00	100.00	100.00		
ESAT-6/ Median spot number	39.00	38.00	34.00		7.061/0.029
ESAT-6/(Spot number interquartile range)	19.00,73.00	20.00,82.00	15.00,67.00		
CFP-10/ Median spot number	30.00	42.00	36.00		5.315/0.070
CFP-10 (Spot number interquartile range)	11.00,67.00	14.00,84.00	11.00,76.75		

TB: tuberculosis; PTB: pulmonary TB; PETB: pulmonary and extrapulmonary TB; EPTB extrapulmonary TB

## Discussion

Overwhelming evidence has suggested that morbidity, misdiagnosis and missed diagnosis of TB has remained at high levels over the past few years. This is primarily due to the prevalence of acquired immune deficiency syndrome (AIDS), growing population mobility, and increasing *M. tuberculosis* drug-resistant mutants [13, 14]. Although there has been a variety of approaches for diagnosing *M. tuberculosis*, the sensitivity and specificity of these assays is not satisfactory, posing enormous challenges for clinical diagnosis [15]. Fortunately, T-SPOT.*TB* is a new, promising diagnosis method, that has been broadly applied in clinical research. This test detects IFN-γ released from T-cells that are exposed to the *M. tuberculosis*-specific antigens ESAT-6 and CFP-10, to measure T-cell number and determine *M.TB* infection status [16]. In addition, this method shows satisfactory sensitivity (78.4%) and greater specificity (59.0 to 93.0%) than previous assays [17–20]. Moreover, a previous study has highlighted that active TB, LTBI, and old TB can all be detected using this method [21]. Another study has revealed that specificity is correlated with LTBI and old TB [22]. Here, our work aimed to explore the practical application effectiveness of T-SPOT.*TB* across various TB types and locations and analyze this assay’s performance in a large dataset.

Our findings suggest that T-SPOT.*TB* positive tests are distributed in different age groups, and that positive rates increase with age. A plausible explanation for this finding is that the probability of exposure to *M.TB* increased with age, which increased the risk for TB. This further validated the fact that there is a large population of patients with LTBI and old TB in China. The number of LTBI (4612 cases) was higher than active TB (1814 cases), which is consistent with other research [23]. Additionally, previous investigations have demonstrated that the T-SPOT.*TB* technique is also successfully employed in specimens other than blood such as pleural fluid, ascitic fluid, and cerebrospinal fluid [24]. Furthermore, the test results of these specimens provided relatively higher diagnostic value than a whole blood test [23]. However, Keng et al. stated that T-SPOT.*TB* values for other specimen types remain to be determined, because of numerous uncertainties and large bias [25].

There were 29 clinical departments in our analysis. Although the highest inspection rate emerged in the respiration department (8261 cases), followed by the rheumatology department (2924 cases), the thoracic surgery department (53.20%) displayed the highest positive rate. Moreover, the results showed that there were 13 departments where the positive rate was higher than the respiration department. In addition, we found that active TB (1814 cases) included PTB (547 cases), PETB (547 cases), and EPTB (1068 cases) groups, suggesting that EPTB showed a predominant infection rate. It is because EPTB cases were characterized by complicated etiologies, atypical clinical presentations, difficulties in diagnosis, and therapy and irreversible damage, which will lead to the high infection rate.

Notably, we first conducted a spot number analysis of T-SPOT.*TB* tests in terms of TB infectious types and locations in three TB groups, including active TB, LTBI and old TB. The results suggested that the number of spots in ESAT-6 and CFP-10 wells were not significantly different between each disease group, but the composition rate in each group was dramatically different in ESAT-6 and CFP-10 double positive microwells (*p* < 0.01). Additionally, we found that the spot number and composition rate were statistically different among the three groups. In this study, we highlighted that ESAT-6 and CFP-10 tests were independent, while spot numbers in each disease group were not statistically different. Therefore, we inferred that there was no significant difference in subjects’ responses to the two specific antigens. In addition, comparative analyses among the three groups implied that the spot number in active TB was higher than LTBI and old TB. Many researchers have claimed that although the positive correlation between spot numbers and TB activity has not been determined, increased spot counts are observed when TB activity was stronger [26, 27], an observation that is consistent with our results. We thus infer that higher spot counts can distinguish active TB from LTBI and old TB. In addition, previous work has evaluated the application values of T-SPOT.*TB* in detecting PTB and EPTB, and found that negative T-SPOT.*TB* results could be considered vital predictors for TB diagnosis [28]. However, the relationship between spot number and these two types of TB (PTB and EPTB) has not been investigated [29, 30]. Here, we found that there were no significant differences in the number of spots and composition rates for intra-or inter-subgroups analyses (*p* > 0.01), which suggests that the identification of PTB, PETB, and EPTB on the basis of the spot number level is not satisfactory.

There were several limitations in this work. Previous studies have pointed out that the immune status of subjects may affect the results of T-SPOT.*TB*. In this study, we did not consider numerous pivotal factors associated with the patients’ immune states, including T-cell activity, nutritional status, additional complications, body mass index, and smoking and drinking. This likely created a mixed basis for results and may influence further stratified analyses in results of T-SPOT.*TB*. Moreover, a comprehensive analysis that combines T-SPOT.*TB* test results, TB etiology, imaging, and histopathology with area-specific TB prevalence is still needed for TB detection and prevention.

## Conclusions

In conclusion, T-SPOT.*TB* test proved to be valuable for distinguishing between LTBI, active TB and old TB. In addition, a higher spot number level was observed in the active TB patient group, but T-SPOT.*TB* test showed low specificity for the diagnosis of different active TB subtypes.

## Data Availability

The datasets used during the current study available from the corresponding author on reasonable request.

## Abbreviations

AIDS: Acquired immune deficiency syndrome
CFP-10: Culture filtrate protein 10
EPTB: Extrapulmonary TB
ESAT-6: Early secreted antigenic target 6
LTBI: Latent TB infection
PETB: Pulmonary and extrapulmonary TB
PTB: Pulmonary TB
TB: Tuberculosis
